# Cloud computing for detecting high-order genome-wide epistatic interaction via dynamic clustering

**DOI:** 10.1186/1471-2105-15-102

**Published:** 2014-04-10

**Authors:** Xuan Guo, Yu Meng, Ning Yu, Yi Pan

**Affiliations:** 1 Department of Computer Science, Georgia State University, 34 Peachtree Street, Atlanta, USA

**Keywords:** Cloud computing, Genome-wide association studies, Dynamic clustering

## Abstract

**Backgroud:**

Taking the advan tage of high-throughput single nucleotide polymorphism (SNP) genotyping technology, large genome-wide association studies (GWASs) have been considered to hold promise for unravelling complex relationships between genotype and phenotype. At present, traditional single-locus-based methods are insufficient to detect interactions consisting of multiple-locus, which are broadly existing in complex traits. In addition, statistic tests for high order epistatic interactions with more than 2 SNPs propose computational and analytical challenges because the computation increases exponentially as the cardinality of SNPs combinations gets larger.

**Results:**

In this paper, we provide a simple, fast and powerful method using dynamic clustering and cloud computing to detect genome-wide multi-locus epistatic interactions. We have constructed systematic experiments to compare powers performance against some recently proposed algorithms, including TEAM, SNPRuler, EDCF and BOOST. Furthermore, we have applied our method on two real GWAS datasets, Age-related macular degeneration (AMD) and Rheumatoid arthritis (RA) datasets, where we find some novel potential disease-related genetic factors which are not shown up in detections of 2-loci epistatic interactions.

**Conclusions:**

Experimental results on simulated data demonstrate that our method is more powerful than some recently proposed methods on both two- and three-locus disease models. Our method has discovered many novel high-order associations that are significantly enriched in cases from two real GWAS datasets. Moreover, the running time of the cloud implementation for our method on AMD dataset and RA dataset are roughly 2 hours and 50 hours on a cluster with forty small virtual machines for detecting two-locus interactions, respectively. Therefore, we believe that our method is suitable and effective for the full-scale analysis of multiple-locus epistatic interactions in GWAS.

## Background

Genome-wide association study (GWAS) has been proved to be a powerful genomic and statistical inference tool, and its goal is to identify genetic susceptibility through statistical tests on associations between a trait of interests and the genetic information of unrelated individuals [1]. In genetics, genotype-phenotype association studies have established that single nucleotide polymorphisms (SNPs) [2], one type of genetic variants, are associated with a variety of diseases [3]. However, the primary analysis paradigm for GWAS is dominated by the analysis on susceptibility of individual SNPs, which accordingly can only explain a small part of genetic causal effects for complex diseases [4]. For better understanding underlying causes of complex disease traits, identifying joint genetic effects (epistasis) across the whole genome has attracted more attentions [5]. As a matter of fact, single locus-based approaches are insufficient to detect all interacting genes, especially for those with small marginal effects. The term epistasis was first used in 1909 and it was referred as an extension of the concept of dominance for alleles within the same allelomorphic pair [6]. In recent literatures, epistasis has been defined generally as the interaction among different genes [7]. Many studies [8-11] have demonstrated that the epistasis is an important contributor to genetic variation in complex diseases such as asthma, breast cancer [12], diabetes, coronary heart disease [13], and obesity [14]. In this article, we consider epistatic interactions as the statistically significant associations of *t*-SNP modules (*t*≥2) with phenotypes [15], i.e. the full association in terms of logistic regression.

Recently, the problem of detecting high-order genome-wide epistatic interaction in GWAS has attracted extensive research interests. There are two challenges in finding high-order genome-wide epistatic interaction on large GWAS dataset [16]: The first arises from heavy computational burden, i.e. the number of association patterns increases exponentially as the order of interaction goes up. For example, 1.25×10^11^ statistical tests are required to detect pairwise interactions for a dataset with 500,000 SNPs. The second challenge is that existing approaches lack statistical powers for searching high-order multi-locus models of disease. Many computational algorithms have been proposed to overcome two preceding difficulties. They can be broadly categorized to three groups: exhaustive search, stepwise search and heuristics approaches.

The naive solution to tack the problem is exhaustive search using *χ*^2^ test, exact likelihood ratio test or entropy-based test for all modules of multiple-locus. Marchini et al. [5] showed that it was computationally possible to test two-locus associations allowing for interactions in GWAS based on current computation resources. Examples in exhaustive search, like MDR and its extensions, utilize repeated cross-validations and permutation tests to evaluate accuracy and significance of classification [7,17]. In addition, Wan et al. [18] proposed a boolean operation-based representation to speed up the collection of contingency tables [19,20]. One major barrier for exhaustive search is the intensive computation, and thus parallel computing was adopted to further speed up the analysis of gene-gene interactions. For example, GBOOST [21] is a GPU framework based implementation of BOOST, and PIAM [22] is developed by Liu et al, which used the multi-thread to perform Genome-wide interaction-based association (GWIBA) analysis for exhaustive two-locus searches. However, finding higher order (more than 2 loci) disease-related associations are too computationally expensive to be feasible, especially for large GWAS datasets with millions SNPs. In order to deal with the huge computation request, stepwise search strategies select a subset of SNPs or combinations of SNPs based on some low-order statistic tests (or measures), then extend them to higher order multi-locus interactions if it is statistically possible [5,20,23]. Stepwise approaches are much faster than exhaustive algorithms and make high-order genome-wide epistasis finding feasible, but they lose powers when complex diseases show no or little marginal effects. Unlike the previous two strategies, heuristic methods adopt machine learning or stochastic procedures to search the space of interactions rather than explicitly enumerating all combinations of SNPs. SNPruler [24], BEAM [25], epiMODE [26] and epiForest [27] fall into this category. SNPRuler and a few other pattern-based methods use some data mining approaches as filters to reduce the number of SNP combinations without assumptions of models. Based on the Markov chain Monte Carlo (MCMC) theory, BEAM iteratively calculates the posterior probability that a locus is associated with the disease and/or involved with other loci in epistasis interactions. EpiMODE first uses the Gibbs sampling strategy with a reversible jump Markov chain Monte Carlo procedure to simulate the posterior distribution that genetic variants belong to the epistatic modules and screens out statistically significant modules based on hypothesis testing. EpiForest treats SNP markers as categorical features, adopts the random forest to discriminate cases against controls, and selects a small set of candidate SNPs that could minimize the classification error. An drawback of heuristics approaches is that they will leave out a great deal of significant interactions which can be reported by first two searching strategies.

In this paper, we provide a cloud based computational method, named “Dynamic Clustering for High-order genome-wide Epistatic interactions detecting” (DCHE), to address above challenges. Taking advantages of recent high-performance computing (HPC) technologies – cloud computing to accelerate computations, DCHE adopts an elaborated dynamic clustering procedure to maximize statistic significance for SNP combinations and ranks top ones as results. One benefit of cloud computing technologies is that the executional environment and experimental conditions can be easily and completely customized by newbies in distributed computing, even for large distributed infrastructures [28]. Furthermore, since the infrastructure is rented on a pay-per-use rule, immediate access to required resources for scientific experiments become possible without planning beforehand. With cloud computing, DCHE conducts statistic tests on merged groups of genotype categories determined by the dynamic clustering. Each grouped genotype category tends to share a similar effect associating with corresponding phenotypes. Truly disease-related joint genetic effects will gain higher ranking values, if genotype combinations can be correctly clustered together. Systematic experiments on simulated two- and three-locus disease models datasets show that DCHE is more powerful in finding epistatic interactions than some recently proposed methods including TEAM [29], SNPRuler, BOOST and EDCF [20]. Our experiments on two real genome-wide case/control datasets, Age-related macular degeneration (AMD) and Rheumatoid arthritis (RA) demonstrate that DCHE is feasible for the full-scale analyses of multi-locus associations on large GWAS datasets and it enriches a great deal of novel, significant high-order epistatic interactions which have not been reported in literatures.

## Results and discussion

We first give definitions of 6 simulated disease models and the power metric used to evaluate the effectiveness of DCHE in comparison with other 4 popular epistatic interactions detecting methods, i.e. TEAM [29], SNPRuler [24], EDCF [20], BOOST [18]. Three reasons for choosing above 4 approaches are as follows: (1) TEAM, EDCF and BOOST all use the exhaustive search strategy for detecting two-locus interactions, so the comparison of their performance is fair; (2) a recent review tested five available methods and recommended BOOST and TEAM as a powerful tool for searching epistatic interactions on a genome-wide scale [15]; (3) our goal is to discover high-order epistatic interactions from GWAS data, and among 4 detectors excluding DCHE, only SNPRuler and EDCF are equipped the ability to search interactions with more than 2 SNPs. Before experiments on simulated datasets, a discussion on how to control the false positive rate is illustrated because the Bonferroni correction, most common method for controlling error rate, can be too conservative to filter significant interactions. We also present results of DCHE on two real GWAS dataset, Age-related macular degeneration (AMD) and Rheumatoid Arthritis (RA). Interactions detected by DCHE from different orders demonstrate a great number of novel, potentially disease-related genetic factors. At the end, a systematic performance evaluation of DCHE’s cloud implementation is conducted on a standard Windows Azure cloud cluster with up to 40 small size Virtual Machine (VM) instances. The speed-up achieved by DCHE shows an approximately theoretical acceleration when the cardinality of epistatic interaction increases.

### Experimental design

#### Data simulation

To evaluate the effectiveness of DCHE, we perform extensive simulation experiments using six disease models with two- and three-locus associations. The unassociated SNP genotypes is generated by the same procedure used in previous studies [18]. Minor allele frequencies (MAFs) are uniformly sampled from the set [0.05,0.5]. By assuming Hardy-Weinberg equilibrium, we can sample the genotype $g_{i}^{j}$ for individual *j*. For embedded disease models, 4 two-locus epistasis models and 2 three-locus epistasis models are chosen by given odds tables or penetrance table which can be found in Additional file 1: Tables S1–S3, and named these six models from model 1 to 6. In addition, we conduct tests on 50 two-locus epistasis models without marginal effects as BOOST and EDCF did in [18,20]. For models 1 to 4 and 50 models without marginal effect, each simulated dataset contained *M*=1000 SNPs and *N*= 800 or 1600 with balanced samples in case and control under each parameter setting. For model 5, one dataset has 1000 SNPs and 2000 or 4000 samples with *N*^*U*^=*N*^*D*^. For model 6, *M*=2000 and *N* is reduced to 400 and 800 with balanced cases and controls.

A disease model can be defined either by specifying the penetrance table or the odds table. Relations among penetrance *p*(*D*), odds ${\text{ODD}}_{g_{i}}$ and the probability *p*(*D*|*g*_*i*_) that an individual will be affected with a given genotype combination *g*_*i*_ can be calculated as Equation 1, 2.

(1) $$\begin{array}{ll} {\text{ODD}}_{g_{i}} & =\frac{p\left(D|g_{i}\right)}{p\left(\bar{D}|g_{i}\right)}=\frac{p\left(D|g_{i}\right)}{1-p\left(D|g_{i}\right)}\; \end{array}$$

(2) $$\begin{array}{ll} p\left(D|g_{i}\right) & =\frac{{\text{ODD}}_{g_{i}}}{1+{\text{ODD}}_{g_{i}}}\; \end{array}$$

Following [18], the disease prevalence *p*(*D*) and genetic heritability *h*^2^ are given by Equation 3, 4.

(3) $$\begin{array}{ll} p\left(D\right) & =\underset{i}{\sum }p\left(D|g_{i}\right)p\left(g_{i}\right)\; \end{array}$$

(4) $$\begin{array}{ll} h^{2} & =\frac{\underset{i}{\sum }{\left(p\left(D|g_{i}\right)-p\left(D\right)\right)}^{2}p\left(g_{i}\right)}{p\left(D\right)\left(1-p\left(D\right)\right)}\; \end{array}$$

For simplicity, we adopt same parameters as used in [18] for model 1 to 4, i.e. *p*(*D*)=0.1, *h*^2^=0.03 for model 1 and *h*^2^=0.02 for Models 2, 3 and 4, MAF ∈{0.1,0.2,0.4}. For model 5, we adopt similar setting in [20], i.e. *p*(*D*)=0.1, effect size *λ*=0.2, *β*∈{4,1.5,1,0.7,0.5} and MAF ∈{0.1,0.2,0.3,0.4,0.5}. For model 6, MAFs of disease associated loci are fixed to 0.5. Effect parameters *α* and *θ* in odds tables for all six models are determined numerically using same procedures in [25]. Settings for 50 models without marginal effect are similar to [30], i.e. *h*^2^ ranges from 0.05 to 0.4 with five intervals and MAF equals to 0.2 or 0.4.

#### Statistical power

In the comparison of performances on simulated data, 100 datasets are generated for each setting. In one dataset, we embed one ground-truth epistatic interaction. The measure of discrimination power used in [18] is adopted, which is defined as the fraction of 100 datasets on which only top interaction given by the method matches the ground-truth. For all programs, the ground-truth interaction are detected if it is set to the most significant one and its adjusted p-value is larger than the critical value which is setted to 0.1 in following experiments.

#### Experimental setting

Programs, TEAM, SNPRuler, BOOST (64 bit) and EDCF are downloaded from websites provided by their authors. For experiments on simulations, DCHE (in Java), SNPRuler (in Java) and BOOST are conducted on a 64-bit Windows 8 platform with 1.8 GHz Intel CPU and 4 GB RAM; since TEAM and EDCF only provided executable program on Linux platform, experiments for TEAM and EDCF are conducted on a 64-bit Linux platform with 2.3 GHz AMD CPU and 16 GB RAM. Experiments on two real datasets are performed on Windows Azure platform with up to 40 small size VMs.

### False positive rate

Since DCHE uses stepwise strategy similar to EDCF, we also adopt two levels of multiple comparisons: (1) test Mt combinations for *t* loci for a dataset with *M* SNPs; (2) test dynamic clustering results, which could end with up to 3^*t*^ possible genotype combinations with *d* groups, *d*∈{3,4,5,6}. If we use the Bonferroni correction for above two level multiple tests, the upper bound of possible ways to do combination is Mt63t. Hence, it is too conservative to obtain significant interaction modules. In order to reasonably loose the strictness, inspired by EDCF we combine the Bonferroni correction and permutation tests for these two levels, that is Bonferroni corrections for *t* loci combinations and permutation tests for the dynamic clustering procedure. More specifically, the significant level for an epistatic interaction is calculated as Equation 5.

(5) α=α0/Mt

**Figure 1:**
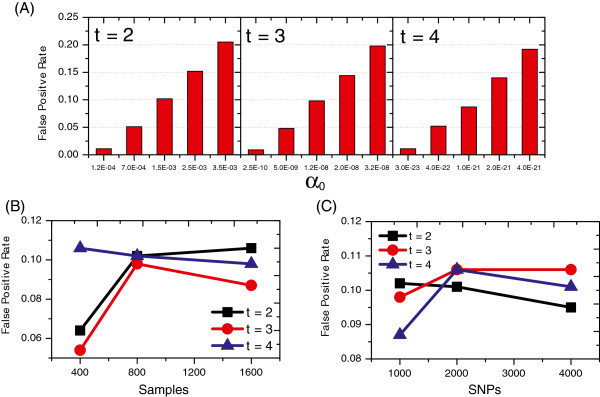
**False positive rates under null models.** The plot in **A** shows the false positive rates of DCHE using different *α*_0_s for different *t*s, and the plots in **B** and **C** show the false positive rates of DCHE for different *t*s when sample size and the number of SNPs vary.

In Equation 5, *α*_0_ is estimated from permutation tests for different *t*s on null simulations and Mt represents the Bonferroni correction. To properly control the false positive rate, we simulated datasets with five different settings for each *t*, i.e. we either fix *M*=1000 and set *N* to 400, 800 and 1600 or fix *N*=800 and set *M* to 1000, 2000 and 4000. Note that one thousand datasets are generated under one setting. The false positive rate is defined as *n*_*false*_/1000, where *n*_*false*_ is the number of datasets where DCHE has found one or more interaction modules. Test results shown in Figure 1 illustrate: for a general setting of critical level 0.1, a recommended *α*_0_ is 1.5×10^−3^ for two-locus disease model detection, 1.2×10^−8^ for three-locus disease model detection and 1.0×10^−21^ for four-locus disease model detection. In addition, the false positive rates tend to decrease or remain nearly unchanged as the number of samples and SNPs go up (Figure 1B and 1C). Therefore, in tests of simulated datasets and two real GWAS datasets, we set *α*_0_=1.5×10^−3^,1.2×10^−8^,1.0×10^−21^ for *t*=2,3,4, respectively, to control the overall false positive rate for DCHE, unless otherwise stated.

### Two-locus disease models

**Figure 2:**
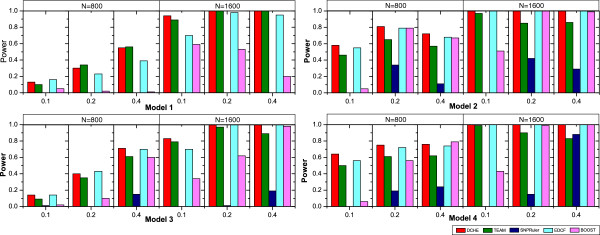
**Performance comparison of DCHE, TEAM, SNPRuler, EDCF and BOOST on disease models 1–4.** Performance comparison of DCHE, TEAM, SNPRuler, EDCF and BOOST on four disease models for different allele frequencies and sample sizes. The red, green, blue, cyan and magenta bars show powers of DCHE, TEAM, SNPRuler, EDCF and BOOST, respectively. Models are ordered from top to bottom and from left to right and they are model 1, model 2, model 3 and model 4.

For a fair comparison, interactions reported by all programs are filtered using the critical value 0.1 as the significant threshold. Test results are illustrated in Figure 2 for model 1 to 4. An common trend for all programs is that power is increasing as sample size increases from 800 to 1600. Most methods show more power when ground-truth model interactions’ MAFs are larger, except that BOOST shows less power on model 1 and 2 when MAFs goes up. We can see that DCHE achieves highest or comparable powers on almost all datasets. More specifically, with 24 parameter settings for four disease models, DCHE outperforms other four methods at 9 settings and obtains full powers at 10 settings and gains comparable results at 5 settings. Taking results from datasets with *N*=1600 for example, it is obvious that DCHE defeats other approaches with nearly 100% powers. For a more straight comparison, we introduce a new concept, the overall quality *q*=*n*_*correct*_/*n*_*total*_, where *n*_*correct*_ is the number of datasets where programs successfully detect the ground-truth interactions and *n*_*total*_ is the total number of datasets. When *N*=800,*M*=1000, the overall quality for DCHE, TEAM, SNPRuler, EDCF and BOOST are 0.541, 0.455, 0.087, 0.508 and 0.31, respectively. When *N*=1600,*M*=1000, all five programs achieve higher accuracies than former settings and *q* are 0.981, 0.912, 0.162, 0.944 and 0.681, respectively. Note that DCHE, TEAM and EDCF have abilities to achieve more than 90% powers, and powers for DCHE reach to at least 98% on datasets with 1600 samples. Note that BOOST is designed to identify significant statistical interaction without considering the main effects, so it is reasonable that our method DCHE and other two methods, i.e. EDCF and TEAM, outperform BOOST for detecting the model 1 through 4. The reason why we still put the BOOST into the experiments is that the biologists might be more interested in epistatic interaction as long as it shows significant association genotypes with phenotypes. In addition, similar designs of experiments can be found in other literatures [15,18,20].

**Figure 3:**
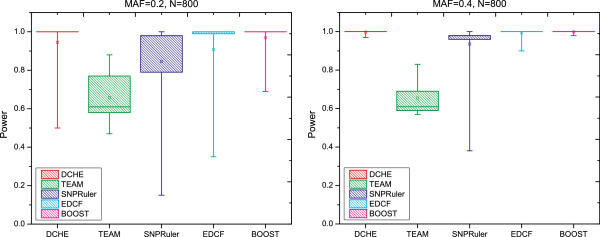
**Performance comparison on 50 models without main effects.** For each model, we simulate data using sample size 800 and *MAF*∈{0.2,0.4}. The red, green, blue, cyan and magenta boxes show powers of DCHE, TEAM, SNPRuler, EDCF and BOOST, respectively.

Moreover, we conduct tests on 50 disease models with little marginal effects. For convenience, penetrance tables for 50 models are not listed, and they are available in literature [30]. Since most methods gain near full powers, we use box plots to demonstrate overall performances in Figure 3. We can see that DCHE, EDCF and BOOST achieve comparable results in two subfigures. Specifically, they can accurately detect embedded associated SNPs interactions under most settings. On the contrary, TEAM and SNPRuler lose significant powers on both datasets with *MAFs*=0.2 or 0.4. A common trend to previous experimental results is that five methods tend to possess more powers as *MAFs* increase. After carefully examining results from five techniques, we can find that DCHE apparently outperforms other three methods except BOOST, although the difference is not too much. A possible explanation is that these embedded models with little main effects are more suitable for model-based detection strategy, and DCHE is a model-free based method. If we adopt the same overall quality defined in previous paragraph to evaluate performances, *q* are 0.972, 0.656, 0.891, 0.951 and 0.984 for DCHE, TEAM, SNPRuler, EDCF and BOOST, respectively.

### Two-locus additive models

Since we intend to find statistically significant associations of *t*-SNP interaction (*t*≥2) with phenotypes, an additive model is used to evaluate method for detecting additive effects. The additive model with 3 different settings can be found in Additional file 1: Table S4. The cell in the table is the odd-ratio of given genotype, and the odd-ratio can be accumulated as the presence of minor allele(s). Both the additive effects of two disease related alleles are equal, which equal to *β*. For the simulation process, we adopt the one which is used in [18], and the value of *α* and *β* are determined by the prevalence *p*(*D*) and the genetic heritability *h*^2^. We fix *p*(*D*)=0.1 and *h*^2^=0.03, set *MFA*=0.1,0.2 and 0.4 by giving *N*=800 and 1600. We simulate 100 datasets under each setting (3 pairs of *α* and *β*) with 1000 SNPs under 800 and 1600 samples in balance.

Test results are illustrated in Figure S2 in Additional file 1 for the additive models. As we expect, DCHE, TEAM and EDCF show limited powers when the sample size is small, and the power goes up as the size of sample increases. SNPRuler and BOOST can not detect any pairs, since BOOST is designed to detect the statistic interaction which is absent in the additive model. Following our preceding measurement, the overall qualities are 0.850, 0.858 and 0.771 for our method, TEAM and EDCF, respectively. It is worthy to note that TEAM is time consuming compared to DCHE. Under the same computing environment, TEAM takes about 2 hours to finish analysis of 100 simulated datasets with 1600 samples, while it can be done in 15 minutes by using DCHE.

### Three-locus disease models

**Figure 4:**
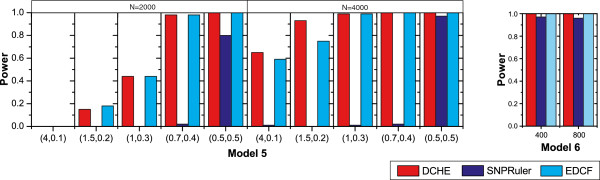
**Performance comparison on the three-locus epistasis models.** Performance comparison of DCHE, SNPRuler and EDCF on two three-locus epistasis models, model 5 and model 6, for different allele frequencies and sample sizes. The red, blue and cyan bars show powers of DCHE, SNPRuler and EDCF, respectively.

For comparisons on three-locus disease models, two methods are dropped, i.e. TEAM and BOOST, because both of them are designed only for detecting two-locus interactions. Based on settings of model 5 given in previous sections, we get 10 groups of datasets with 100 replicates, which can be further categorized to two families with *N*= 2000 or 4000. Note that when *MAF*=0.5, there is no marginal effect, otherwise disease models have *λ*=0.2. Experimental results are illustrated in Figure 4. Similar to models 1 to 4, three programs generally tend to get more powers as *MAF* or sample size increases. Considering parameter *β* in (*β*,*MAF*), we can see that powers go up when *β* goes down for all methods. A significant difference can be found is that SNPRuler can only obtain acceptable results when (*β*=0.5,*MAF*=0.5); otherwise SNPRuler hardly gets powers. Although the distinction between DCHE and EDCF is not too much, we can still observe that DCHE hits more ground-truth SNPs interactions than EDCF does at datasets with *N*=4000. Additionally, overall qualities for DCHE and EDCF are 0.514 and 0.52 with *N*=2000, and the overall quality for DCHE rises up to 0.914 comparing with 0.866 for EDCF.

For Model 6, we set *MAF*=0.5 and population prevalence *p*=0.01 as EDCF does in [20]. Note that model 6 with penetrance table in Additional file 1: Table S6 gets the maximum *h*^2^ when $p\in (0,\frac{1}{16}]$. Three methods’ results are shown in Figure 4, from which we can see that all methods can get nearly full powers for model 6. With considering the overall quality, DCHE and EDCF both reach 100% and SNPRuler hits to 0.965.

### Experiments on AMD data

Age-related macular degeneration (AMD) is an acquired degeneration of the retina which usually affects older adults and results in a loss of vision in the centre of the visual field. Like many other chronic diseases, AMD is caused by a combination of genetic and environmental risk factors, including and not limited to macular degeneration gene, too much exposure to sunlight and smoking. The reported AMD dataset contains genotypes of 103,611 SNPs from 96 affected individuals and 50 controls [31]. Before applying DCHE on AMD dataset, the same quality control used in [18] is applied: SNPs with more than 10% missing data or *MAF*<0.05 or p-values from Hardy-Weinberg Equilibrium (HWE) tests less than 0.001 are removed. Subsequently, 90,449 SNPs from 50 controls and 96 cases are left in the dataset. The setting of parameters for DCHE is as follows: *l*={10000,4000,2000}, *t*=2,3,4 and *α*_0_=1.5×10^−3^,1.2×10^−08^,1.0×10^−21^ for two-, three- and four-locus interactions detections, respectively.

When we set *α*_0_=1.5×10^−3^ to filter out insignificant interactions for two-locus epistatic interactions detecting, DCHE can hardly report any qualified modules, so we select top- *k* modules to conduct analysis. For *t*=3 and 4, DCHE generates more than 1000 pairs epistatic interactions. In order to give a straight view of results, we introduce two concepts, “centre SNPs” and “centre genes”, similar to those in [32]: we arrange those SNPs and genes in descending manner according to their frequencies showing in top- *k* interactions, and select top- *s* SNPs or genes as “centre”. Based on the previous procedure, Table 1 and Table 2 give a general view of DCHE’s results on AMD dataset with *k*=1000 for *t*=2, 3, *k*=500 for *t*=4, and *s*=6. Names of SNPs or genes showed in boldface indicate their first time to appear in the table. As we can see, top- *k*- *s* SNPs or genes tend to share some common elements among different settings of order of interactions, *t*. For AMD dataset, two SNPs (rs380390 and rs1329428) already have been reported as disease associated SNPs with AMD based on results from single allelic association tests with *df*=1 [31]. In our results, DCHE also ranks rs380390 and rs1329428 as top 2 centre SNPs both in two- and three-locus epistatic interactions detecting. Both rs380390 and rs1329428 locate inside the gene CFH whose location is 1p32, and their protein products have an essential role in the regulation of complement activation and restricting the innate defence mechanism to microbial infections. In addition to rs380390 and rs1329428, we also find another interesting SNP, rs3781868, in the category *t*=4. rs3781868 locates in the gene NPAT with location 11q22-q23 which is known to be essential for histone mRNA 3’ end processing and recruiting CDK9 to replication-dependent histone genes.

**Table 1:** Centre SNPs identified in top-1000/500 SNPs interactions on AMD dataset

**# SNPs per**	**Centre SNPs from analyses of AMD dataset**	
**interaction**	**Centre SNPs (Genomic position)**	**# Interacting SNPs**
	**rs380390 (Ch1: 196701051)**	524
	**rs1329428 (Ch1: 196702810)**	253
2	**rs1394608 (Ch5: 155783294)**	23
	**rs1740752 (Ch10: 38538771)**	20
	**rs1363688 (Ch5: 174609731)**	11
	**rs10512174 (Ch9: 88886574)**	11
	rs380390 (Ch1: 196701051)	709
	rs1329428 (Ch1: 196702810)	106
3	rs1363688 (Ch5: 174609731)	63
	**rs618499 (Ch11: 108148839)**	47
	**rs1926489 (Ch13: 92667989)**	35
	**rs3781868 (Ch11: 108059569)**	34
	rs380390 (Ch1: 196701051)	459
	rs618499 (Ch11: 108148839)	188
4	rs3781868 (Ch11: 108059569)	115
	**rs294278 (Ch3: 31127911)**	36
	**rs300780 (Ch2: 110819)**	35
	**rs315511 (Ch1: 84849116)**	28

Note: SNP in boldface indicates the first time to appear in the table.

**Table 2:** Centre genes identified in top-1000/500 SNPs interactions on AMD dataset

**# Genes per**	**Centre genes from gene-only SNP analyses**	
**interaction**	**Centre genes**	**# Interacting genes**
	**CFH: complement factor H**	777
	**ZNF25: zinc finger protein 25**	23
2	**SGCD: sarcoglycan, delta (35kDa dystrophin-associated glycoprotein)**	23
	**LRIG3: leucine-rich repeats and immunoglobulin-like domains 3**	14
	**DRD1: dopamine receptor D1**	11
	**ISCA1: iron-sulfur cluster assembly 1**	11
	CFH: complement factor H	815
	DRD1: dopamine receptor D1	63
3	**ATM: ataxia telangiectasia mutated**	47
	**GPC5: glypican 5**	43
	**NPAT: nuclear protein, ataxia-telangiectasia locus**	34
	**KDM4C: lysine (K)-specific demethylase 4C**	25
	CFH: complement factor H	459
	ATM: ataxia telangiectasia mutated	191
4	NPAT: nuclear protein, ataxia-telangiectasia locus	115
	LRIG3: leucine-rich repeats and immunoglobulin-like domains 3	73
	**TGFBR2: transforming growth factor, beta receptor II (70/80kDa)**	38
	**ACP1: acid phosphatase 1, soluble**	35

Note: Gene in boldface indicates the first time to appear in the table.

We also analyse results using gene-only from top-1000/500 SNPs subset. Top-1000/500 SNPs are mapped to disease-related genes, which have been annotated on the HuGE Navigator database, and we get 720, 851 and 424 genes for *t*=2,3,4 showed in Table 3. It is obvious that the majority of centre genes have not yet been reported by HuGE as associated with the AMD disease. Applying similar analysis used in [32], we submit centre genes reported in Table 3 to the ToppGene, a candidate gene prioritization tool [33], to evaluate biological significances of these novel genes. From DCHE’s results, ToppGene enriches an cell-cell communication pathway with the name ‘REACTOME_ADHERENS_JUNCTIONS_INTERACTIONS’. Reported in Reactome, this pathway contains 14 centre genes, and only one gene in this pathway presents in HuGE Navigator database. As gene names given in HGNC, these genes are PVRL3, CDH18, CDH10, CDH11, CDH12, CDH13, CDH2, CDH4, CDH7, CDH6, CDH9, CDH8, CADM1, CADM3.

**Table 3:** The disease association of DCHE selected genes from gene-only SNP analyses

		**Reported in HuGE**	
**# SNPs per**	**# DCHE genes in top**	**Navigator database**	
**interaction**	**1000 (500) SNP pairs**	**# Analyzed**	**# DCHE**
		**genes**	**genes**
2	720	151	20
3	851		28
4	424		13

For the detection of two-locus epistatic interaction on AMD dataset, DCHE successfully indentifies rs380390 and rs1329428 reported by the original paper. Comparing with results obtained by other existing methods, we find that there are some overlaps between them. For example, DCHE lists two pairs of SNPs with ranking 246 and 247 (rs1394608 and rs3743175, rs1394608 and rs2828155), which have been identified by epiMODE applied on AMD dataset [26]. In addition, DCHE reports another interaction module (rs1394608 and rs6847164), whose *p*−*value* is more significant than the above two (*p*−*value*_*unadjusted*_=6.78×10^−10^). rs1394608 resides within the intron of SGCD, a gene located on chromosome 5q33-34, which has been implicated in AMD [26]. rs6847164 resides within PDE5A, a gene located on chromosome 4q27. According to the databases of NCBI and Entrez, PDE5A is involved in the regulation of intracellular concentrations of cyclic nucleotides and is important for smooth muscle relaxation. DCHE has also detected other significant three-locus and four-locus interaction modules: (rs10487833, rs10495593, rs1740752) and (rs9302001, rs10497231, rs380390, rs1940041) whose unadjusted p-values are 3.24×10^−18^ and 8.28×10^−28^, respectively. rs10487833 locates about 0.3Mb upstream of gene NAMPT on chromosome 10. NAMPT encodes a protein which is thought to be involved in many important biological processes, including metabolism, stress response and aging. rs10497231 resides at about 0.3Mb downstream of gene KCNH7 on chromosome 2. KCNH7 encodes a member of the potassium channel, voltage-gated, subfamily H related to the functions including regulating neurotransmitter release, heart rate, insulin secretion, neuronal excitability, epithelial electrolyte transport, smooth muscle contraction, and cell volume. rs9302001 locates about 0.4Mb upstream of gene ABCC4 on chromosome 13. The protein encoded by ABCC4 is a member of the superfamily of ATP-binding cassette (ABC) transporters, which is thought to play a role in cellular detoxification as a pump for its substrate, organic anions. The clustering details of genotype combinations of the above three interaction modules can be found in Additional file 1.

### Experiments on RA data

Rheumatoid arthritis (RA) is a chronic and systemic autoimmune disorder, which causes that afflicted joints become warm, swollen, tender, stiff, and in the final stage, deformed. RA is believed to be a heterogeneous disease in which genetic factors account for 60% of disease susceptibility by rough estimation. The genome-wide RA data comes with 545,080 SNPs, 868 cases and 1194 controls and it is collected by the NARAC and provided by the Genetic Analysis Workshop 16. The same quality control for AMD dataset has also been applied to RA dataset. Subsequently, 487,678 SNPs from 1,194 controls and 868 cases remained. The parameters for DCHE are set as follows: *l*={10000,4000,2000} for *t*=2,3,4 and *α*_0_=1.5×10^−3^,1.2×10^−8^,1.0×10^−21^ for two-, three- and four-locus interaction detection, respectively.

Based on definitions of centre SNPs, Table S9 in Additional file 1 gives a general view of DCHE results on RA dataset with *k*=1000 for *t*=2,3,4 and *s*=10. From the overview of centre SNPs, we can see that most top-ranked SNPs are coming from chromosome 6 at which the well known MHC region locates. Recent studies conducted by the WTCCC via single-locus association mapping have shown that RA are strongly associated with the MHC region [18]. In addition to SNPs in chromosome 6, some other interesting SNPs located at other chromosomes are detected in 4-order SNPs interactions, including rs888206 and rs1359679. rs888206 locates in the gene MMD, monocyte to macrophage differentiation-associated, whose protein product is expressed in vitro differentiated macrophages but not freshly isolated monocytes. Another suggested alternative function of MMD is related to an ion channel protein in maturing macrophages. rs1359679 locates near the gene BRINP1 on chromosome 9 with location 9q32-q33. According to NCBI, BRINP1 is within a chromosomal region which shows loss of heterozygosity in some bladder cancers and it may undergo hypermethylation-based silencing in some bladder cancers. Table S10 in Additional file 1 lists a summary of top-ranked reported genes from HuGE Navigator database and DCHE. Similar to results in AMD dataset, the majority of potentially disease-related interacting genes detected by DCHE are novel for RA dataset. Through analyses of top-10 frequent centre genes in Table S11 of Additional file 1, we can see that some genes already have been established associations with RA, including HLA, BTNL2, C6orf10, and we also find some other potential genetic causal factors, like CCAR2, KDM4C.

### Computation time

From a practical point of view, a key issue of detecting high-order epistatic interactions in GWAS is the computational efficiency. In this section, we evaluate the performance of the proposed parallel strategy on Windows Azure cloud platform with respect to its speed-up. To measure the speed-up, we keep the size of datasets constants and increase the number of nodes (computing cores) in the cloud system. Speed-up given by the larger system is defined by the following formula [34]:

(6) $$\text{Speedup}\left(p\right)=\frac{T_{1}}{\text{Tp}}$$

where *p* is the number of nodes (computers), *T*_1_ is the execution time on one node and *T*_*p*_ is the execution time on *p* nodes. The ideal parallel method is expected to demonstrate linear speed-up: a system with *p* computing nodes generates a speed-up of *p*. However, linear speed-up is only a theoretical predication because the speed-up is curved by both the inevitable node failures and the communication cost which increase as the number of computing nodes in cluster becomes large.

**Figure 5:**
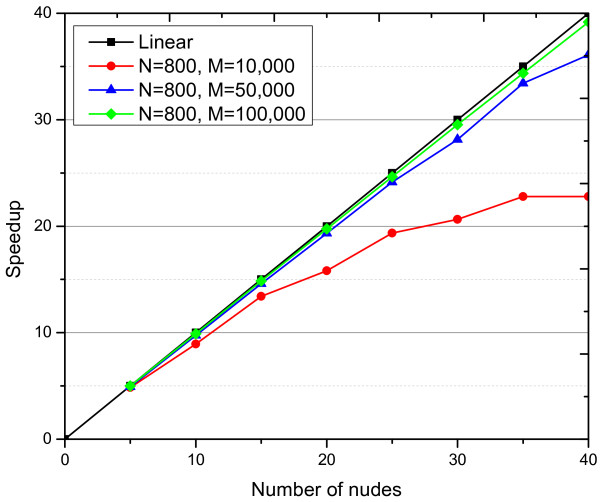
**Speed-up.** Computing nodes are sampled from 1 to 40 with 5 as interval. The red, blue, cyan and grey curve show functions of speed-up of *M*=10,000, *M*=50,000 and *M*=100,000 with sample size fixed to 800.

We perform the speed-up evaluation on datasets with quite different sizes ranging from 10,000 to 100,000. The number of nodes (virtual machines provided by Windows Azure platform) varies from 1 to 40 with 5 as minor units. Figure 5 shows speed-up for these datasets with three settings. As results showed, the proposed cloud implementation of DCHE has a very good performance with respect to the speed-up. When there are a limited number of SNPs in the dataset, e.g. *M*=10,000, it has a lower speed-up curve, because dataset with such size along SNP dimension can be easily processed by a stand-alone version program alone in a couple of minutes. As the size of datasets increases, speed-up performs better (green and blue curves). Therefore, we believe our proposed parallel strategy for DCHE on Windows Azure cloud computing platform can be used to treat massive data analyses efficiently.

### Discussion

#### Relationship between DCHE and BOOST

Two key differences lie between DCHE and BOOST: 

•BOOST is designed to detect statistic interactions via log-linear models, using the *χ*^2^ test with *df*=4. DCHE aims to identify significant associations via model-free method, using the *χ*^2^ test with *df* ranges from 2 to 6.

•BOOST can be only applied to find gene-gene interaction within two-locus. DCHE is flexible to search *t*-SNP interaction patterns with *t*≥2 and has been implemented in cloud platform on Windows Azure.

In order to prove that our method possesses the power to detect epistatic interaction which demonstrates significant interaction effect, the overlap of results from BOOST and DCHE on two-locus simulated models are showed in Additional file 1: Figure S3. The bar with full height means that all modules produced by BOOST are all marked as the most significant interaction by DCHE. For models embedded ground-truth modules with both main and interaction effects, nearly all modules reported by BOOST are also found by DCHE. Only 3 out of 24 do not reach 100%, but they are all above 90%. For datasets without main effect or with weak main effect, although 7 out of 50 do not get 100% overlap, it is still convincing to conclude that significant interactions identified by BOOST can be mostly covered by DCHE.

#### The advantages and limitations of DCHE

The development of DCHE is triggered by the limitations of existing works on finding high-order epistatic interaction from genome-wide data. DCHE displays several advantages over existing methods: 

1. DCHE detects high-order epistatic interactions from genome-wide data without exhaustive enumeration;

2. DCHE is a model-free method and does not assume any prior distribution;

3. DCHE does not assume any particular epistasis model. This is very important for real studies because the patterns of SNP interactions are generally unknown and could be very complex;

4. DCHE provides a list of ranked interaction based on their significance.

The current version of DCHE cannot distinguish which type of epistatic interaction contributing most to the significant SNP module, i.e. the statistical interaction or the full association with only main effects. It is a general problem for other existing model-free methods, like MDR, BEAM, TEAM, SNPRuler and EDCF. Future work can be extended to addressing the above issue. In addition, how to reduce false positive errors is a challenging problem in GWASs for our method, although we combine permutation test and Bonferroni correction to control type I error. Incorporating the haplotype information and pathway information to further help reduce false positive errors can be another direction of our future work.

## Conclusions

In this manuscript, a cloud based algorithm DCHE, for detecting high-order genome-wide epistatic interactions is proposed. The key step of DCHE is the dynamic clustering procedure, which is used to guide on how to merge genotype categories to a limited and variable number of groups. By dynamic clustering, DCHE tries to approximately categorize genotypes with similar genetic effects on phenotypes. The cloud implementation of DCHE takes advantages of cloud computing technology, especially the Windows Azure cloud platform with high level and efficient I/O operation, queue and blob storage, to guarantee the correctness and enable parallel statistic testing. Comprehensive and systematic comparisons on simulated datasets shows that DCHE can obtain more or comparable powers for both two- and three-locus interaction modules detecting comparing to other four recently developed algorithms, i.e. TEAM, SNPRuler, EDCF and BOOST. Furthermore, experiments on two real datasets of AMD and RA demonstrate that DCHE discovers many novel high-order associations which are significantly enriched in cases and a great deal of centre SNPs and genes which only appear in detections of high order epistatic interactions. The computation time analysis confirms that our method provides a promising way to accurately accelerate large genome wide association studies.

## Methods

### Notation

Suppose a GWAS dataset has *M* diallelic SNPs and *N* samples. In general, bi-allelic genetic markers use uppercase letters (e.g. *A*, *B*,...) to denote major alleles and lowercase letters (e.g. *a*, *b*) to denote minor alleles. For encoding three genotypes, one popular way is to use {0,1,2} to represent {*AA*,*Aa*,*aa*}, respectively. In a GWAS case-control dataset, *N*^*U*^ denotes the number of cases (i.e. disease individuals) and *N*^*D*^ denotes the number of controls (i.e. normal individuals). *X* is utilized to indicate the ordered set of the *M* SNPs, and *X*_*i*_ represents the *i*-th SNP in *X*. *MAF*(*X*_*i*_) denotes the minor allele frequency of *X*_*i*_ and $g_{i}^{j}$ denotes the genotype of *j*-th individual at *X*_*i*_. For *t*-locus interaction, $\left(X_{i_{1}},\ldots ,X_{i_{t}}\right)$, one genotype combination denotes as $\left(g_{i_{1}},\ldots ,g_{i_{t}}\right)$.

### Dynamic clustering

An intuitive strategy to detect genome-wide epistatic interactions is to test differences of genotypes’ frequencies for single SNP or SNPs’ combinations in cases and controls. The contingency table in Table 4 gives an example for two-locus disease model, where there are 9 genotype combinations and *N*^*U*^=*N*^*D*^=800. Numbers within the parentheses are counted from controls. Cells with higher frequencies in cases are coloured by grey background. Some methods, like Multifactor dimensionality reduction (MDR) [12] and its extensions [35], take the case/control ratio of each genotype combination to test associations between SNP combinations and disease status. However, the frequency cannot be a fair indicator to uncover disease related associations, because it can be biased by many factors, including effect size, allele frequency, linkage disequilibrium between markers and disease loci as well as sampling errors. Other recent developed strategies use Pearson’s *χ*^2^ test, exact likelihood ratio test and entropy-based test to examine the independence of observations. For the example in Table 4a, the unadjusted p-value from Pearson’s *χ*^2^ test with 8 degrees of freedom is 1.724×10^−18^. If we use Bonferroni correction to adjust p-value, this pair can still be significant with threshold 0.05 for a large GWAS dataset. However, it will not always be the case, and some limitations, including uneven or insufficient samples, tiny penetrance on single genotype, would dramatically affect the adjusted p-value. Another toy example sampled from a two-locus multiplicative effects model (see Table 5) is shown in Table 4b, where *N*^*U*^=*N*^*D*^=400. Normally, the approximation to the *χ*^2^ distribution breaks down if more than 20% expected frequencies below 5. The unadjusted p-value of Table 4b is 1.09×10^−6^ and the adjusted p-value is 0.547 if *M*=1,000, which is obviously larger than 0.05. Another popular method, BOOST [18] which utilizes the likelihood ratio to test statistic, cannot get a significant result for Table 4b by setting the significant level to 0.1. We can observe the same result by applying EDCF which utilizes the concept of frequent item to group genotype combinations and adopts the *χ*^2^ test with *df*=2.

**Table 4:** Examples for the contingency tables

	**(a)**			**(b)**		
	**BB**	**Bb**	**bb**	**BB**	**Bb**	**bb**
AA	71(108)	97(151)	44(47)	40(55)	49(76)	13(21)
Aa	89(138)	184(184)	**93(55)**	43(62)	**110(103)**	**49(22)**
aa	29(43)	**113(57)**	**80(17)**	16(24)	**50(27)**	**30(10)**

Note: The entries in boldface are the ones with ratios greater than $\frac{N^{D}}{N^{U}}$.

**Table 5:** Two-locus interaction multiplicative effects

	**BB**	**Bb**	**bb**
AA	*α*	*α*	*α*
Aa	*α*	*α*(1+*θ*)	*α*(1+*θ*)^2^
aa	*α*	*α*(1+*θ*)^2^	*α*(1+*θ*)^4^

To address the preceding issues, we propose a dynamic clustering procedure. Basically, we first merge all $3^{n^{t}}$ genotype cells to *n*^*d*^ groups based on certain combination criteria, where *n*^*d*^ ranges from 3 to 6. The criterion to combine two genotype categories is rooted from their similar effects which associate with phenotypes. Secondly, we collect statistic test values on merged groups. For better illustration, we take Table 5 for example. Although there are 9 genotype categories, some have same penetrances, so we can partition them into 4 groups, where penetrances are *α*,*α*(1+*θ*),*α*(1+*θ*)^2^ and *α*(1+*θ*)^4^. In reality, it is different to predict the order of complex disease model and its penetrance table. Therefore, we try to find a statistically significant evaluation of interactions in a stepwise manner by merging genotype categories into a range of number of groups and test levels of significance. We select the most significant one as the evaluation for *t* SNPs interaction. The full algorithm of dynamic clustering is as follows. 

•Step 1. For a set of SNPs, cross-tabulate genotype combinations of SNPs with the categories of the dependent variable (phenotype).

•Step 2. Find a pair of genotype combinations whose 2×2 sub-table is least significantly different. If this significance does not reach a critical value, merge the two combinations and consider this merger as a single compound combination, and repeat this step.

•Step 3. Calculate the significant evaluation for each merged group pattern when categories’ number is larger than three and less than six. Select the most significant one as the unadjusted p-value as the evaluation for the current interaction.

In Step 2, there are several ways to measure the difference of a 2-by-2 contingency table, like Pearson’s *χ*^2^ test with *df*=1 and phi coefficient. In our algorithm, we adopt Pearson’s *χ*^2^ test with *df*=1 to measure the difference. Following the dynamic clustering procedure, we can get the most significant group pattern as 161, 129, 110 in cases and 238, 59, 103 in controls for Table 4. Note that the *df* varies when the number of clusters changes. According to our setting that *n*^*d*^ ranges from 3 to 6, the *df* changes from 2 to 5, respectively. Along the clustering, we calculate the *p*−*value*_*unadjusted*_ for each clustering with corresponding *df*. The trace of merging for Table 4b is (0, 1, 2, 3, 6), (5, 7, 8) and (4), if cells are labelled from left to right, from top to bottom and start from 0. It is easy to output this ground-truth interaction by the combination of Bonferroni correction and permutation tests for controlling type-I error. (*p*−*value*_*unadjusted*_=1.15×10^−9^ and the significant level is *α*=3.0×10^−9^ with false positive rate nearly equals to 0.1).

### Evaluation of interactions

The goal of DCHE is to identify *t*-SNP (*t*≥2) epistatic interactions significantly associated with phenotype. As stated in [7,18], epistasis can be interpreted as the statistical interaction or the full association. The evaluation of interactions used by DCHE is similar to detect full associations in model-based methods. In terms of logistic regression, the epistatic interaction we are looking for may contain main effects or interaction effects or both. In order to detect the significant association between genotype and phenotype, we use model-free method and p-value from Pearson’s *χ*^2^ test to indicate the significance. Since DCHE aims to find high-order genome-wide epistatic interaction, the high-order interaction module may consist of one or some redundant SNPs, which do not contribute to increase the significance. To avoid such cases, we give a definition of the least possible significant epistatic interaction.

#### **Definition1**

A SNPs module $\left(X_{i_{1}},X_{i_{2}},\ldots ,X_{i_{t}}\right)$ is considered as the least possible significant epistatic interaction by giving the significant level *α*, if it meets the following two conditions: 

(1) the *p*−*value* of clusters of $(X_{i_{1}},X_{i_{2}},\ldots ,X_{i_{t}})\leq \alpha$;

(2) the *p*−*value* of clusters of any subset of $(X_{i_{1}},X_{i_{2}},\ldots ,X_{i_{t}})<$ the *p*−*value* of clusters of $\left(X_{i_{1}},X_{i_{2}},\ldots ,X_{i_{t}}\right)$.

### Algorithm

Since testing all high-order SNP combinations is impossible for large GWAS datasets of millions of SNPs, we utilize a stepwise strategy to emulate and run dynamic clustering on all two-locus SNPs combinations. As shown in a recent theoretical study [36], the possibility that a high-order (size- *t*) combination with strong differentiation shows zero differentiation in all of its subsets decreases dramatically when *t* increases (generally it becomes impossible for *t*≤5). Therefore, we use top- *l*_*t*_ low-order SNPs combinations which demonstrate some significance as candidates. For higher order, we add one SNP *X* each time to interactions and re-invoke the dynamic clustering procedure, until *t* reaches the defined value. We adopt same bitwise operations and Boolean Representation as introduced in BOOST [18] to collect and compress contingency tables. The details of the sequential algorithm is shown in Algorithm 1. The cloud implementation of DCHE will be elaborated in next section.

Each column in matrix *M* is converted to 3 rows in matrix *W* based on Boolean Representation (line 1–2). An ascending list where redundancy is not allowable is initialized with size max(*l*). The structure of an element in *L* consists a pair of key and value that the key is SNPs combinations and value is the unadjusted p-value (line 3). DCHE uses bitwise operations to collect contingency tables for all two-locus interaction and calculates evaluations of significance via DynamicClustering procedure. The p-value will be inserted into *L* (line 4–9). DCHE only selects ${\text{top-}l}_{t^{\prime }}$ interactions to extend in DynamicClustering procedure and inserts estimated significance into another candidate list *L*^′^. At the end of each iteration for specific *t*, list *L* gets cleaned and DCHE transfers ${\text{top-}l}_{t^{\prime }}$ elements from *L*^′^ to *L* and new list $L_{t^{\prime }}$ (line 10–23). When *t* reaches the defined value, top- *l*_*t*_ interaction modules with *p*−*value*>*α* will be written into the result file (line 24–33).

The time complexity of dynamic clustering is *O*(3^*t*^). According to the theory in [36], we only need to apply dynamic clustering procedure for up to 4 order of SNPs combinations. So when *t*=2, the time complexity to test all 2-locus interactions is *O*(*NM*^2^). Inserting an element into ascending list takes time *O*(log(max(*l*))). The total time complexity for 2-locus interaction detection is *O*(*NM*^2^)+*O*(log(max(*l*))). When *t*=3, the time complexity to extend all candidate 2-locus interactions to 3-locus modules is *O*(*l*_3_*M*). Hence, the entire time complexity reaches to *O*(*l*_3_*M*)+*O*(*NM*^2^)+*O*(log(max(*l*))), if the user plans to search 3-locus interaction. Similar time complexity analysis can be applied to higher order interaction detection by using our DCHE.

### Cloud implementation

**Figure 6:**
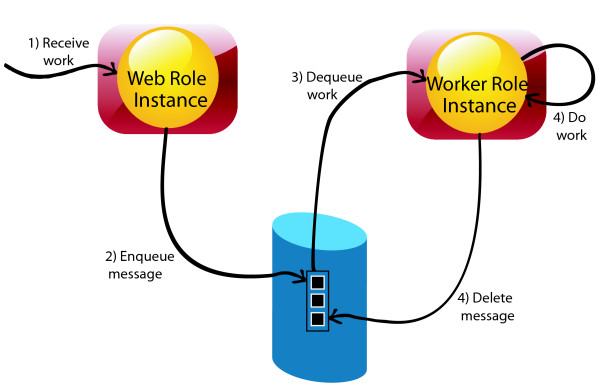
Illustration of the suggested Windows Azure application model.

We implemented DCHE on the Windows Azure platform [37]. Due to several practical considerations of association detection for GWAS, like typical GWAS datasets reaching up to size of gigabytes, statistic tests for all SNPs combinations. A Windows Azure application running in the cloud or in data centre can be divided into logical parts which are called roles in Windows Azure as shown in Figure 6. A role contains a specific set of codes and will be running on relatively independent environment. In addition, Windows Azure applications can be easily deployed to a customized cloud infrastructure, even for users who are not HPC experts.

Since statistic tests of all interaction are independent, it is suitable and easy to parallel dynamic clustering procedure. The details of cloud framework for DCHE is described as follows (see Figure S1 in Additional file 1). Windows Azure storage service come with highly efficient distributed file systems and two basic storage features using in DCHE are as follows. (1) Blob: like traditional file system, where files can be retrieved by its name, and the limit size of single blob file can be up to 50GB [37]. (2) Queue: An asynchronous massage passing mechanism for communication among computing nodes; an important feature of Queue is that messages will automatically show up again until explicitly deleted [38]. Two issues for cloud computing are that it may consumes huge time in communication and it is difficult to balance workload between nodes. Therefore, we design a way to split the whole dataset into several parts and pack up a bunch of dynamic clustering calculations. Detailed steps for Cloud DCHE are elaborated in next paragraph. 

•Step 1. The whole matrix of GWAS dataset is partitioned into *d* parts on SNPs dimension. For the *i*-th portion, it contains $\left\{p^{j},g_{i}^{j},g_{i+d}^{j},\ldots \right\}$, where *j*={1,2,…,*M*} and *p* denotes the phenotype. The reason why we split dataset in such way is that it can optimally balance both workloads within and among partitions.

•Step 2. Each worker role instance reads one copy of all partitions which are compressed using Boolean Representation [18].

•Step 3. Customized parameters used in DCHE will be set through web role instance, including size of the matrix, numbers of partitions of data, critical value, maximum loci interaction pattern and length of the ascending list. Note that the number of worker role instances do not need to be specified because all tasks are executed asynchronously and controlled by a unique file name as a key recognized by worker and master.

•Step 4. A particular work role, named master role, is used to pack tasks commands into a queue and detect running status via emulating files in the blob. There is only one instance of master role, which is programmed to pack a pair of key (unique file name) and value (task orders).

•Step 5. Worker role instances simply fetch commands from queues. The key factor to implement fault tolerant is that same undone task packs will show up again in queues after a user defined period if there are any failure.

•Step 6. All results will be stored in ascending lists and written into blob, when worker role instances finish tasks, i.e. dynamic clustering procedures.

•Step 7. Master role will detect which stage the algorithm is running on and communicate with web role relying on file information in blob.

•Step 8. Web role instance is the interface to interact between user and DCHE by retrieving running status.

## Availability of supporting data

DCHE and its cloud implementation code is available at http://www.cs.gsu.edu/\~{x}guo9/DCHE.html Password for the source code of cloud implementation is dche; The simulated data is available on the original paper authors’ websitel (http://bioinformatics.ust.hk/BOOST.html and http://discovery.dartmouth.edu/epistatic\_data/); The genome-wide Rheumatoid arthritis data is provided by the Genetic Analysis Workshops 16 (http://www.gaworkshop.org/).

## Availability and requirements

Project name: Cloud Computing for Detecting High-Order Genome-wide Epistatic Interaction via Dynamic Clustering; Project home page: http://www.cs.gsu.edu/\~{x}guo9/DCHE.html, and https://sourceforge.net/projects/dche/Operating system(s): Windows 8, Windows Azure Programming language: Java 1.7 or higher; C#, coded in Visual Studio 2012.

## Competing interests

The authors declare that they have no competing interests.

## Authors’ contributions

XG conceived the study, and wrote the manuscript with contributions from other authors. XG designed and implemented the algorithm, DCHE and its cloud version program. YM and NY performed the gene annotation, analyzed the data and critically read the manuscript. YP coordinated the work, conceived and designed the method, and made the major edits. All authors read and approved the final manuscript.

## Supplementary Material

Additional file 1 Supplementary materials. Click here for file
