# Supplementary material for: Cloud computing for detecting high-order genome-wide epistatic interaction via dynamic clustering
Source: BMC Bioinformatics. 2014 Apr 10;15:102. doi: 10.1186/1471-2105-15-102 (PMC4021249; doi:10.1186/1471-2105-15-102)

SUPPLEMENTARY MATERIALS:

Figure S1: The workflow of the cloud implementation of DCHE on Windows Azure platform.

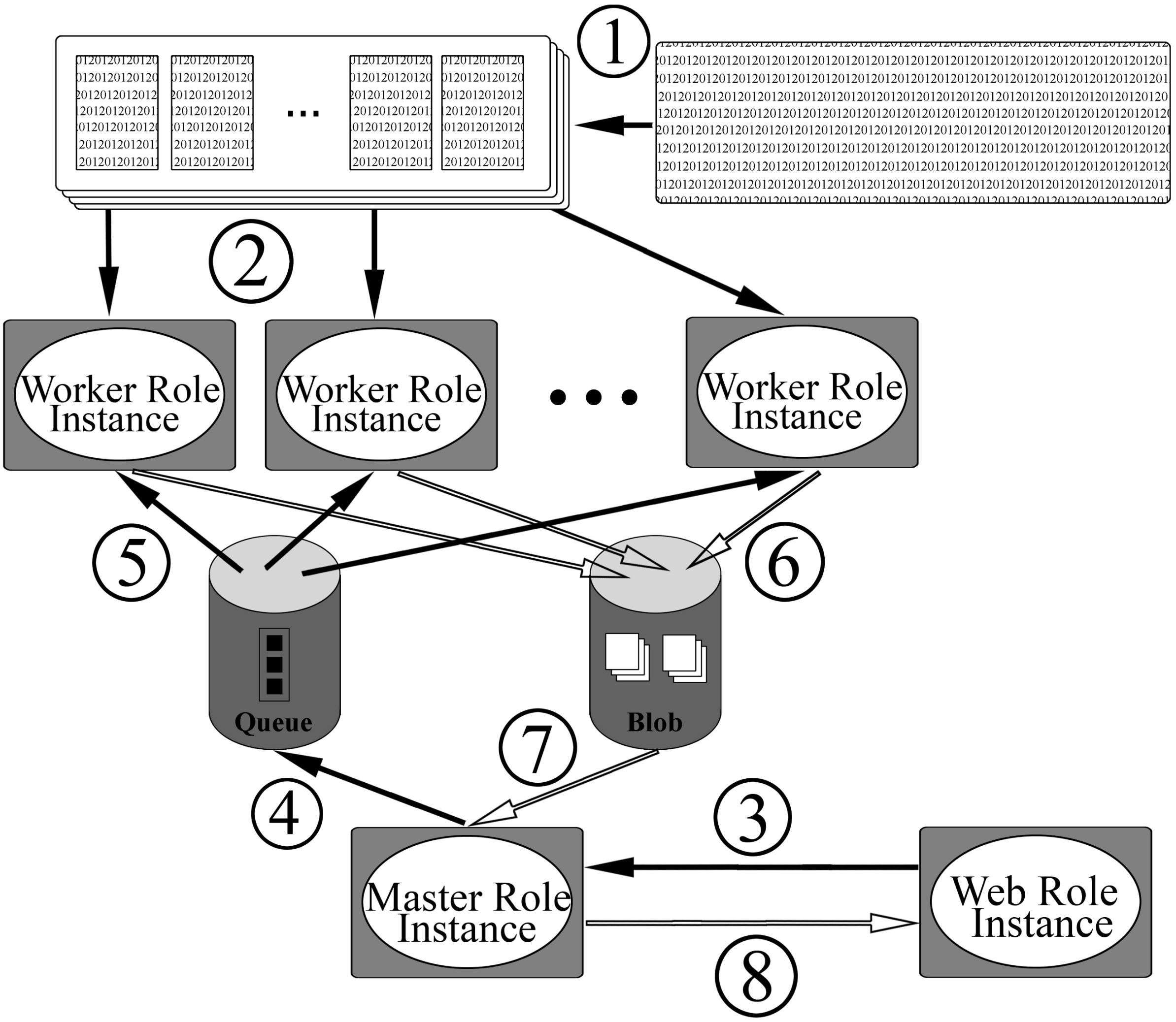

The explanation of steps is in the main text.

Figure S2: **Performance comparison on additive models with main effects.** For each model, we simulate data using sample size 800 and $MAF \in \{0.1, 0.2, 0.4\}$. The red, green, blue, cyan and magenta boxes show powers of DCHE, TEAM, SNPRuler, EDCF and BOOST, respectively.

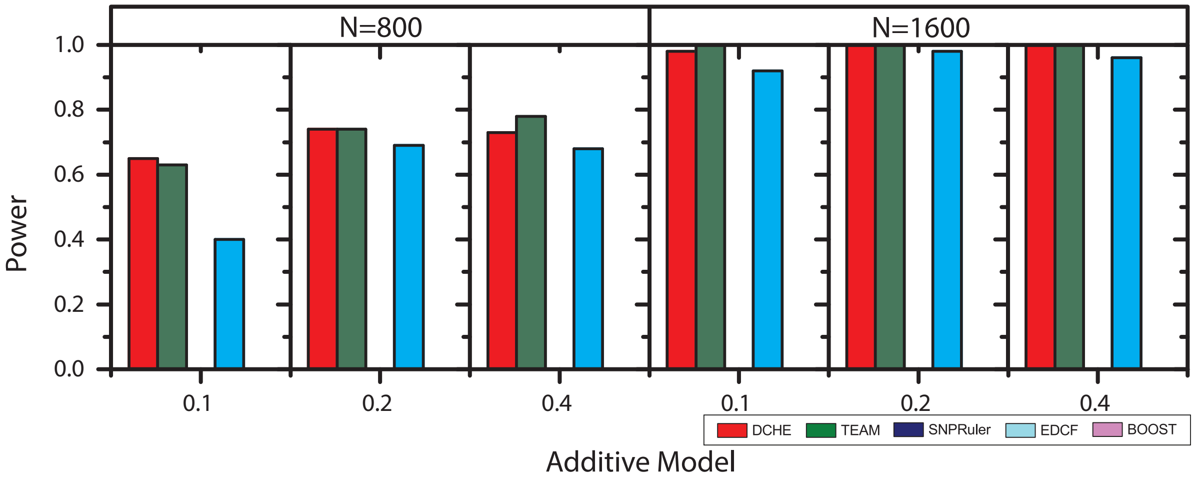

Table S1: Odds tables of epistasis models 1, 2, 3 and 4.

Model 1	BB	Bb	bb
AA	α	α	α
Aa	α	$\alpha(1+\theta)$	$\alpha(1+\theta)^2$
aa	α	$\alpha(1+\theta)^2$	$\alpha(1+\theta)^4$

Model 2	BB	Bb	bb
AA	α	$\alpha(1+\theta)$	$\alpha(1+\theta)$
Aa	$\alpha(1+\theta)$	α	α
aa	$\alpha(1+\theta)$	α	α

Model 3	BB	Bb	bb
AA	α	α	$\alpha(1+\theta)$
Aa	α	$\alpha(1+\theta)$	α
aa	$\alpha(1+\theta)$	α	α

Model 4	BB	Bb	bb
AA	α	$\alpha(1+\theta)$	α
Aa	$\alpha(1+\theta)$	α	$\alpha(1+\theta)$
aa	α	$\alpha(1+\theta)$	α

The parameters α and θ control the prevalence $p(D)$, heritability h^2 by given MAF.

Table S2: Odds table of epistasis model 5.

	BBCC	BbCC	bbCC	BBCc	BbCc	bbCc	BBcc	Bbcc	bbcc
AA	α	α	α	α	α	$\alpha(1+\theta)$	α	$\alpha(1+\theta)$	α
Aa	α	α	$\alpha(1+\theta)$	α	$\alpha(1+\beta\theta)$	α	$\alpha(1+\theta)$	α	α
aa	α	$\alpha(1+\theta)$	α	$\alpha(1+\theta)$	α	α	α	α	α

The parameters α, β and θ control the prevalence $p(D)$, heritability h^2 and marginal effect size λ of disease loci (see definitions in the main text).

Table S3: Penetrance table of epistasis model 6.

	BBCC	BbCC	bbCC	BBCc	BbCc	bbCc	BBcc	Bbcc	bbcc
AA	0	0	16θ	0	0	0	0	0	0
Aa	0	0	0	0	4θ	0	0	0	0
aa	0	0	0	0	0	0	16θ	0	0

With minor allele frequency $MAF = 0.5$, model 6 with this penetrance table reaches the maximum h^2 when penetrance $p \in (0; 1/16]$. There is no marginal effect of a single disease marker.

Table S4: Illustration of odds table of two-locus additive model.

	BB	Bb	bb
AA	α	$\alpha(1+\beta)$	$\alpha(1+\beta)^2$
Aa	$\alpha(1+\beta)$	$\alpha(1+\beta)^2$	$\alpha(1+\beta)^3$
aa	$\alpha(1+\beta)^2$	$\alpha(1+\beta)^3$	$\alpha(1+\beta)^4$

The parameters α, β and θ control the prevalence $p(D)$, heritability h^2 and marginal effect size λ of disease loci (see definitions in the main text).

Table S5: Odds table of two-locus additive models.

	AABB	AABb	AAbb	AaBB	AaBb	Aabb	aaBB	aaBb	aabb	MAF	pD	h^2
1	0.0743	0.1609	0.3486	0.1609	0.3486	0.7552	0.3486	0.7552	1.6359	0.1		
2	0.0603	0.1133	0.2130	0.1133	0.2130	0.7526	0.2130	0.4004	0.4004	0.2	0.1	0.03
3	0.0396	0.0698	0.1232	0.0698	0.1232	0.2175	0.1232	0.2175	0.3837	0.4		

Table S6: The contingency table of (rs1394608 and rs6847164). Here, the $p-value_{unadjusted} = 6.78 \times 10^{-10}$

Clusters	Cases	Controls	Total	Genotypes of rs1394608 and rs6847164
0	80	16	96	10, 11, 20, 22
1	15	29	44	00, 21
2	0	5	5	01, 02, 12
Total	95	46	141	

Table S7: The contingency table of (rs10487833, rs10495593, rs1740752). Here, the $p-value_{unadjusted} = 3.24 \times 10^{-18}$

Clusters	Cases	Controls	Total	Genotypes of rs10487833, rs10495593 and rs1740752
0	77	6	83	002, 010, 102, 111, 112, 122, 201, 211, 212, 220, 221, 222
1	12	15	27	001, 011, 101, 200, 210
2	0	25	25	000, 012, 100, 110, 120, 121, 202
Total	96	46	142	

Table S8: The contingency table of (rs9302001, rs10497231, rs380390, rs1940041). Here, the $p-value_{unadjusted} = 8.28 \times 10^{-28}$

Clusters	Cases	Controls	Total	Genotypes of rs9302001, rs10497231, rs380390 and rs1940041
0	77	0	77	others
1	0	44	44	0111, 0120, 0221, 1000, 1010, 1020, 1111, 1112, 1120, 1201, 1211, 1212, 1220, 1221, 2012, 2120, 2121, 2210, 2220, 2221
2	8	2	10	1100, 1200
3	3	1	4	2112
4	6	1	7	2111
5	2	2	4	2110
Total	96	50	146	

Table S9: Centre SNPs identified in top-1000 SNPs interactions on RA dataset.

#SNPs per interaction	Centre SNPs from analyses of complete SNP set	
	Centre SNPs (Genomic position)	#Interacting SNPs
2	**rs2395175 (Chr6: 32405026)**	880
	**rs660895 (Chr6: 32577380)**	53
	**rs6910071 (Chr6: 32282854)**	17
	**rs2395163 (Chr6: 32387809)**	17
	**rs3763309 (Chr6: 32375973)**	14
	**rs3763312 (Chr6: 32376348)**	14
	**rs7745656 (Chr6: 32680970)**	7
	**rs9275595 (Chr6: 32681355)**	7
	**rs9275374 (Chr6: 32668526)**	6
	**rs9275388 (Chr6: 32669084)**	6
3	rs2395175 (Chr6: 32405026)	866
	rs660895 (Chr6: 32577380)	134
	rs9275595 (Chr6: 32681355)	127
	**rs9275439 (Chr6: 32671521)**	109
	**rs9275427 (Chr6: 32670915)**	103
	**rs9275418 (Chr6: 32670244)**	102
	**rs9275425 (Chr6: 32670874)**	100
	**rs9275555 (Chr6: 32677088)**	99
	**rs9275428 (Chr6: 32670978)**	98
	**rs9275393 (Chr6: 32669439)**	86
4	rs2395175 (Chr6: 32405026)	999
	rs9275595 (Chr6: 32681355)	998
	**rs888206 (Chr17: 53468213)**	100
	**rs1359679 (Chr9: 121597614)**	99
	**rs2817763 (Chr6: 110972494)**	42
	**rs7227652 (Chr18: 46223842)**	41
	**rs2025324 (Chr9: 7102389)**	39
	**rs6577430 (Chr1: 7324468)**	37
	**rs4238618 (Chr16: 12946129)**	34
	**rs3802400 (Chr9: 7119141)**	29

Table S10: The disease association of DCHE selected genes from gene-only SNP analyses.

#SNPs per interaction	# DCHE genes in top 1000 SNP pairs	Reported in HuGE Navigator database	
		#Analyzed genes	#DCHE genes
2	631		77
3	440	793	40
4	719		59

Table S11: Centre genes identified in top-1000 SNPs interactions on RA dataset.

#SNPs per interaction	Centre genes from gene-only SNP analyses	
	Centre genes	#Interacting genes
2	**HLA-DRA: major histocompatibility complex, class II, DR alpha**	890
	**HLA-DQB1: major histocompatibility complex, class II, DQ beta 1**	101
	**BTNL2: butyrophilin-like 2 (MHC class II associated)**	65
	**HLA-DQA1: major histocompatibility complex, class II, DQ alpha 1**	56
	**C6orf10: chromosome 6 open reading frame 10**	33
	**HLA-DQA2: major histocompatibility complex, class II, DQ alpha 2**	29
	**HLA-DRB1: major histocompatibility complex, class II, DR beta 1**	10
	**HLA-DRB9: major histocompatibility complex, class II, DR beta 9 (pseudogene)**	6
	**OR4X1: olfactory receptor, family 4, subfamily X, member 1**	6
	**FSTL5: follistatin-like 5**	5
3	HLA-DRA: major histocompatibility complex, class II, DR alpha	866
	HLA-DQB1: major histocompatibility complex, class II, DQ beta 1	780
	HLA-DQA2: major histocompatibility complex, class II, DQ alpha 2	227
	HLA-DQA1: major histocompatibility complex, class II, DQ alpha 1	134
	**PTPRJ: protein tyrosine phosphatase, receptor type, J**	14
	**FAM84B: family with sequence similarity 84, member B**	13
	**HSD17B8: hydroxysteroid (17-beta) dehydrogenase 8**	11
	C6orf10: chromosome 6 open reading frame 10	10
	**SNX16: sorting nexin 16**	10
	**SNX13: sorting nexin 13**	9
4	HLA-DRA: major histocompatibility complex, class II, DR alpha	999
	HLA-DQA2: major histocompatibility complex, class II, DQ alpha 2	998
	**CCAR2: cell cycle and apoptosis regulator 2**	100
	**MMD: monocyte to macrophage differentiation-associated**	100
	**KDM4C: lysine (K)-specific demethylase 4C**	72
	**CDK19: cyclin-dependent kinase 19**	42
	**CTIF: CBP80/20-dependent translation initiation factor**	41
	**CAMTA1: calmodulin binding transcription activator 1**	37
	**CPPED1: calcineurin-like phosphoesterase domain containing 1**	34
	**PPHLN1: periphilin 1**	32

Figure S3: **Overlap of results from DCHE on ones from BOOST.** The yellow bar is the overlap rate, i.e. what percentage of the top rated modules yielded by BOOST has been detected by DCHE.(A) The overlap percentage on 4 simulated models embedded epistatic interaction demonstrating both main effect and interaction effect. (B) The overlap percentage on 50 models embedded epistatic interaction demonstrating weak main effect, but strong interaction effect.

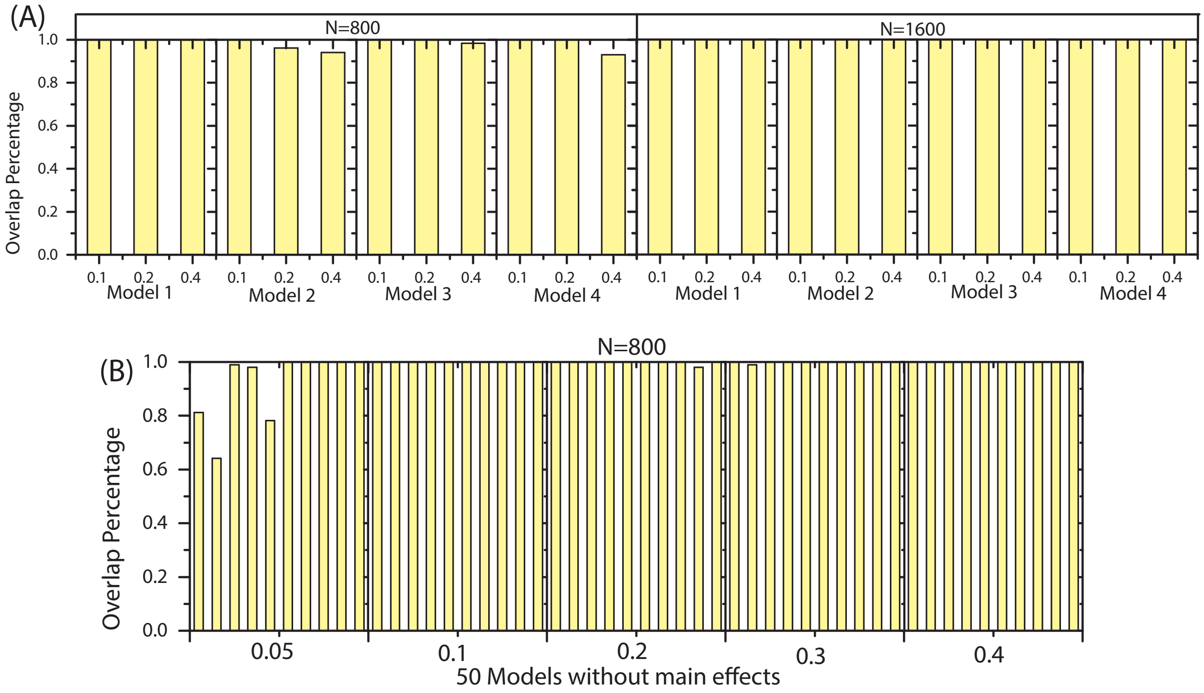

Supplement: Additional file 1 — Supplementary materials. [file 1471-2105-15-102-S1.pdf]
